# Clinical decision trees support systematic evaluation of multidisciplinary team recommendations

**DOI:** 10.1007/s10549-020-05769-1

**Published:** 2020-07-06

**Authors:** Mathijs P. Hendriks, Xander A. A. M. Verbeek, Jeannette G. van Manen, Sannah E. van der Heijden, Shirley H. L. Go, Gea A. Gooiker, Thijs van Vegchel, Sabine Siesling, Agnes Jager

**Affiliations:** 1 Department of Medical Oncology, Northwest Clinics, Wilhelminalaan 12, 1815 JD Alkmaar, The Netherlands; 2 Department of Research and Development, Netherlands Comprehensive Cancer Organization, Utrecht, The Netherlands; 3 Department of Health Technology and Services Research, Technical Medical Centre, University of Twente, Enschede, The Netherlands; 4 Department of Radiology, Northwest Clinics, Alkmaar, The Netherlands; 5 Department of Surgery, Northwest Clinics, Alkmaar, The Netherlands; 6 Department of Medical Oncology, Erasmus MC Cancer Institute, Rotterdam, The Netherlands

**Keywords:** Guidelines, Clinical decision trees, Decision support, Breast cancer, Multidisciplinary team

## Abstract

**Purpose:**

EUSOMA’s recommendation that “each patient has to be fully informed about each step in the diagnostic and therapeutic pathway” could be supported by guideline-based clinical decision trees (CDTs). The Dutch breast cancer guideline has been modeled into CDTs (www.oncoguide.nl). Prerequisites for adequate CDT usage are availability of necessary patient data at the time of decision-making and to consider all possible treatment alternatives provided in the CDT.

**Methods:**

This retrospective single-center study evaluated 394 randomly selected female patients with non-metastatic breast cancer between 2012 and 2015. Four pivotal CDTs were selected. Two researchers analyzed patient records to determine to which degree patient data required per CDT were available at the time of multidisciplinary team (MDT) meeting and how often multiple alternatives were actually reported.

**Results:**

The four selected CDTs were indication for magnetic resonance imaging (MRI) scan, preoperative and adjuvant systemic treatment, and immediate breast reconstruction. For 70%, 13%, 97% and 13% of patients, respectively, all necessary data were available. The two most frequent underreported data-items were “clinical M-stage” (87%) and “assessable mammography” (28%). Treatment alternatives were reported by MDTs in 32% of patients regarding primary treatment and in 28% regarding breast reconstruction.

**Conclusion:**

Both the availability of data in patient records essential for guideline-based recommendations and the reporting of possible treatment alternatives of the investigated CDTs were low. To meet EUSOMA’s requirements, information that is supposed to be implicitly known must be explicated by MDTs. Moreover, MDTs have to adhere to clear definitions of data-items in their reporting.

## Introduction

### Background

The European Society of Breast Cancer Specialists (EUSOMA) recommends that “each patient has to be fully informed about each step in the diagnostic and therapeutic pathway and must be given adequate time to consider the alternatives and make an informed decision” [1]. As diagnostic and treatment modalities in breast cancer are increasing rapidly, clinicians are challenged to apply a growing amount of knowledge during clinical decision-making for optimal patient care. The multidisciplinary team (MDT) is supported by clinical practice guidelines, consolidating knowledge in evidence- or consensus-based recommendations aiming to improve the quality of care [2]. However, as guidelines are increasingly complex and dynamic, it is challenging to overview and consider all relevant recommendations for each clinical decision.

Guideline-based clinical decision trees (CDTs) could be of great value to comply to EUSOMA’s recommendations. To apply CDTs, all relevant data-items for a guideline-based recommendation should be available during MDT meetings and should be reported explicitly. In case the guideline recommendation consists of more than one alternative (e.g., breast surgery vs. preoperative systemic treatment), the MDT should report which alternatives will be proposed to the patient or should be waived substantiated.

In the Netherlands, the Breast Cancer guideline has been set up by a multidisciplinary group of specialists and patients advocates under the auspices of the National Breast Cancer Organization (NABON) [3]. In previous work, we have shown that the Dutch NABON guideline was successfully transformed into 60 clinical decision trees (CDTs) driven by 114 unique data-items, resulting in recommendations for in total 376 unique patient and tumor features combinations [4]. A path through a CDT follows “nodes” that represent patient- and/or disease characteristics (i.e., data-items) and results in “a leaf” representing a guideline recommendation. A CDT therefore defines explicitly which data-items should be minimally available for a guideline-based recommendation.

The main objective of this study is to evaluate the availability of the required data-items during MDT meetings—as verifiable in the electronic health records—for four pivotal CDTs: indication for (1) performing an MRI scan, (2) preoperative systemic treatment (PST), (3) adjuvant systemic treatment (AST) and (4) immediate breast reconstruction (IBR). Our second objectives are (i) to evaluate whether the MDT reports mention multiple alternatives for those cases in which the guideline recommendation consist of more than one alternative; (ii) to evaluate the concordance of recommendations generated by the MDT and the CDTs.

## Method

### Population

This retrospective single-center study was performed in Northwest Clinics, a teaching hospital and oncology center in the province North Holland. All malignancies in Dutch hospitals are registered in the Netherlands Cancer registry (NCR). For this study, all patients aged 18 years or older and diagnosed with breast cancer in Northwest Clinics were selected from the NCR between February 2012 and February 2015 (*N* = 1239). Exclusion criteria were male sex, patients with recurrent breast tumors or advanced breast cancer at diagnosis, patients being treated for other cancer(s) in the past, patients receiving treatment in another hospital and patients who were not at least discussed once in a MDT meeting. A required sample size was calculated to estimate proportions with a 5% accuracy (*n* = *z*2*p(1 − *p*)*d*2, where *n* = sample size, *z* = *z* value for 95% CI 1.96, *p* = largest possible proportion = 0.5 and *d* = accuracy of 5% = 0.05). We expected a dropout rate of 25% based on the exclusion criteria. The required sample size calculated to estimate proportions was 385 patients. Considering the expected dropout rate, 504 patients were randomly selected from the original cohort.

### Guideline-based decision-making using CDTs

CDTs based on the Dutch breast cancer guideline of 2012 were used, which was valid during the study period [4]. For each decision point in the patient care pathway, all applicable guideline recommendations have been synthesized into CDTs. CDT nodes represent patient and disease characteristics (i.e., data-items, such as T-stage) and its branches represent cut-off values (e.g., cT value less than cT2). Every CDT “leaf” represents a guideline recommendation. Each recommendation has one of the following levels: “recommended for” or “recommended against” (a hard recommendation), or “recommended for consideration”. This grading of recommendations to level of evidence is supported by the GRADE approach [5]. CDTs are digitally available in Dutch via a web application (www.oncoguide.nl) and for Android and iOS tablets. Oncoguide can document data output in a standardized, computable data format meeting the FAIR criteria [6].

**Fig. 1:**
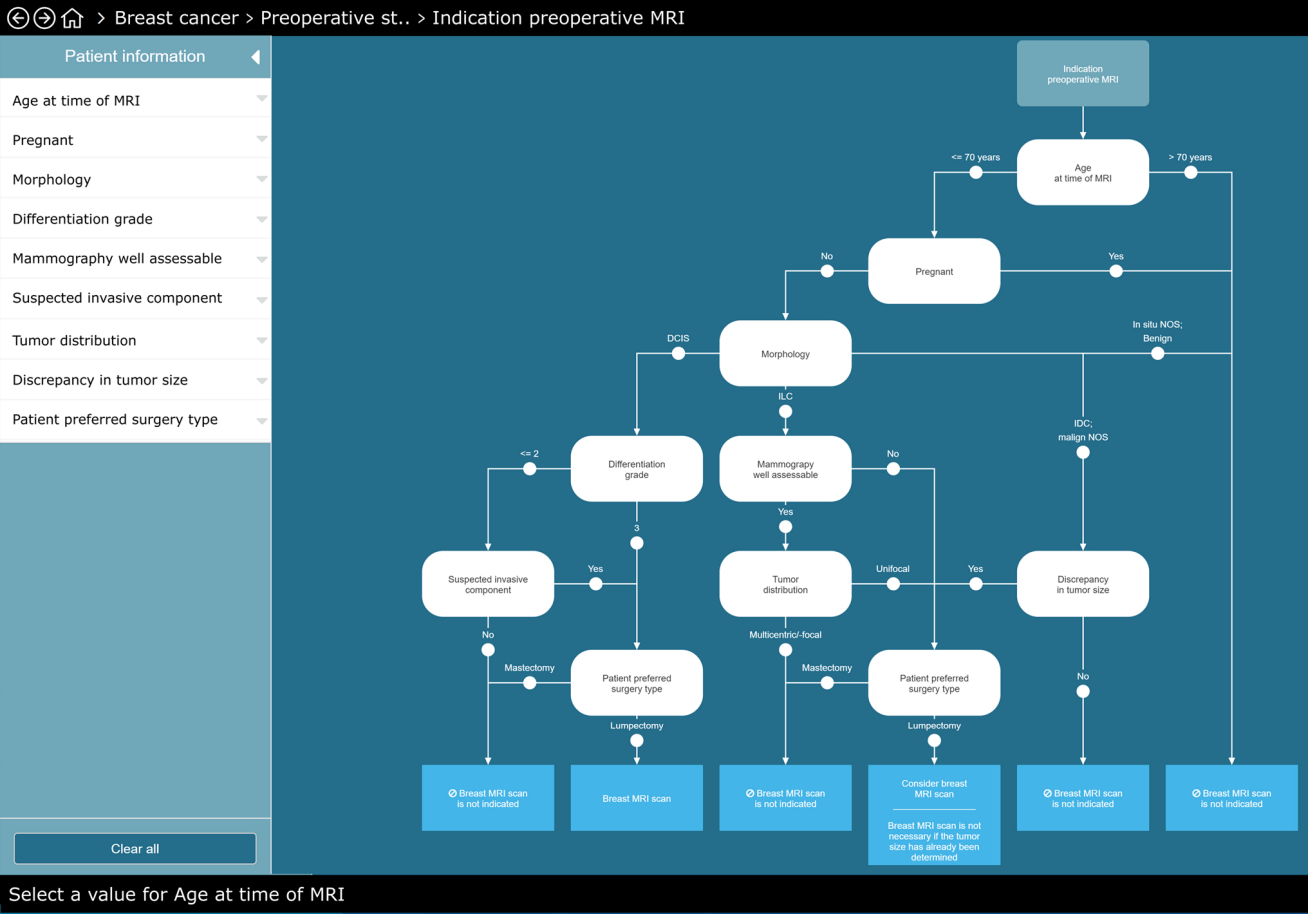
Example of the clinical decision tree (CDT) of “pre-operative MRI scan” in Oncoguide. MRI is indicated in case of (i) breast-conserving surgery, unless tumor size is already assessed; (ii) discrepancy between tumor size assessed by clinical examination, mammography and/or ultrasound; (iii) lobular carcinoma unless unifocal mass on well assessable mammography. **PST = preoperative systemic treatment

**Fig. 2:**
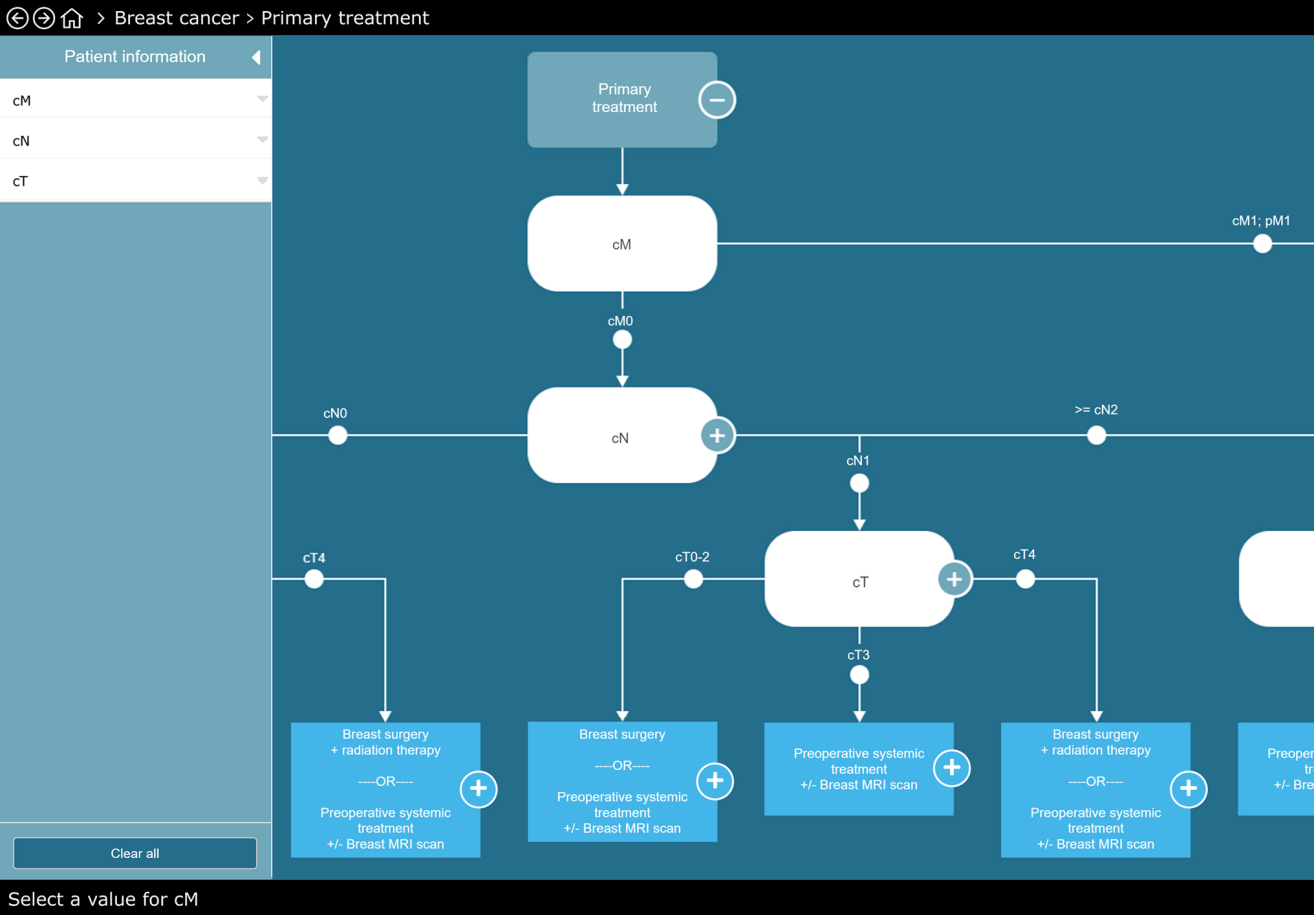
Example of the clinical decision tree regarding first treatment. Note that some “leaves” (i.e., the rectangles at the bottom of the CDT) result in a guideline-based recommendation with more than one alternative

We focused in our study on four pivotal clinical decisions in the care pathway: indication for (1) MRI scan, (2) PST, (3) AST and (4) IBR. These CDTs contain, respectively, six, five, six and four data-items. Fifteen of these 21 data-items are unique. As example we illustrate the CDTs indiction for MRI and first treatment in Figs. 1 and 2.

### Data collection, analysis and availability

For included patients, all data-items needed to complete a path through CDTs in Oncoguide for the associated decision was retrieved retrospectively from the MDT, radiology and pathology reports in the electronic health record independently by two researchers (MH and SH). Data retrieval was restricted to data in the electronic health record as available at the time of the applicable MDT meeting in which each case was discussed. Data on MDT recommendations including explicit consideration of more than one treatment alternative were retrieved from de MDT reports. Concordance of recommendations reported by the MDT and the CDTs was analyzed, including reporting motivations for disconcordance. In case a guideline recommendation was for consideration, it was verified if this was explicitly reported in the electronic health record. Data analysis was performed using Microsoft Excel for descriptive statistics. The dataset generated and analyzed during the current study are not publicly available but are available from the corresponding author on reasonable request.

## Results

Of the 504 randomly selected patients, 110 patients were excluded for the reasons of no invasive breast cancer (*n* = 4), treatment for other cancer(s) in the past (*n* = 58), metastatic disease (*n* = 31), treatment received in other hospitals (*n* = 13), not discussed in at least one MDT meeting (*n* = 3) and not being diagnosed within the research period (*n* = 1). The residual included patients (*n* = 384) were equally divided over the 3 years of study duration (Table 1).

**Table 1:** Patient characteristics of 394 randomly selected cases

	Number	Percentage
Total number	394	
*Age (years)*		
Median	62	
Range	31–93	
> 70 years	104	26
*Period*		
February 2012 till February 2013	127	32
February 2013 till February 2014	134	34
February 2014 till February 2015	133	34
*Tumor type*		
Invasive ductal carcinoma	331	84
Invasive lobular carcinoma	49	12
Other	14	4
*Receptor status*		
ER+ /HER2−	308	78
ER+ /HER2+	13	3
ER−/HER2+	3	1
ER−/HER2−	46	12
Receptor status not available	24	6
*Clinical tumor stage*		
Stage I	215	55
Stage II	156	40
Stage III	23	6
*Pathological tumor stage*		
pCR	11	3
Stage I	172	44
Stage II	149	38
Stage III	35	9
No surgery	27	7

Percentages may not equal 100% due to rounding

*pCR* pathologic complete response

### Availability of data during MDT meetings

Of all required 8004 data-items necessary for the four pivotal CDTs, 808 (10.1%) data-items were missing. Unverifiable data-items were “clinical M-stage” 81.6% (*n* = 659), “assessable mammography” 13.9% (*n* = 112) and 4.6% (*n* = 37) due to missing data on three other items (tumor distribution, ER status and tumor grade).

Data-items as required in the CDT for MRI scan, PST, AST and IBR were complete in 70%, 13%, 97% and 13% of the patients, respectively (Table 2). At maximum, two data-items were missing for each CDT, and this occurred in 1%, 1%, 0% and 2% of patients, respectively. Assuming the most frequent missing data-items “clinical M-stage” and “assessable mammography” as known would result in complete data-item availability in 97%, 99%, 97% and 97%, respectively.

**Table 2:** Availability of data-items during MDT meetings: an analysis using CDTs for four domains in the care path

Indication	Data-item name (values)	Data-item verifiable in EHR	
		No of patients	Percentage
MRI (*n* = 394)			
	Pregnant	394	100
	Age at time MRI (years)	394	100
	Morphology (i.e., lobular carcinoma, ductal carcinoma, other)	394	100
	Mammography well assessable (yes or no)	282	72
	Tumor distribution (not registered, unifocal, multicentric)	383	97
	Discrepancy tumor size: clinical vs. on imaging (no or yes)	394	100
	All data-items available	276	70
Preoperative systemic treatment (*n* = 394)			
	Clinical M-stage* (not registered, cM0 or cM1)	52	13
	Clinical N-stage (not registered, cN0, cN1, cN2, cN3)	394	100
	Clinical T-stage (not registered, cT1a, cT1b, cT1c, cT2, cT3, cT4)	394	100
	Gender (female)	394	100
	ER status (not registered, ER+ , ER−)	390	99
	All data-items available	52	13
Adjuvant systemic treatment (367 patients underwent surgery)			
	Pathologic N-stage (not registered, pN0, pN1, pN2, pN3)	367	100
	N0 risk status		
	Age (years)	367	100
	Pathologic T-stage (not registered, pTis, pT1a, pT1b, pT1c, pT2, pT3, pT4)	367	100
	Tumor grade postoperatively** (not registered, BR gr1, BR gr2, BR gr3)	359	98
	HER2 status postoperatively (not registered, Her2+ , Her2−)	367	100
	ER status*** (not registered, ER+ , ER−)	364	99
	Age (years)	367	100
	All data-items available	356	97
Immediate breast reconstruction (367 patients underwent surgery)^a^			
	Clinical M-stage** (not registered, cM0 or cM1)	50	14
	Clinical N-stage (not registered, cN0, cN1, cN2, cN3)	367	100
	Clinical T-stage (not registered, cT1a, cT1b, cT1c, cT2, cT3, cT4)	367	100
	Tumor distribution (not registered, unifocal, multicentric)	356	97
	All data-items available	46	13

*BR* Bloom Richardson grade

*Clinical M-stage was not explicitly reported, only when staging (PET CT) was performed

**In 7 patients, the pathologist reported that the tumor size was too small for BR grading and in one patient the BR grade was not reported

***In 367 patients, 48 were ER−, 316 ER+ and ER in 3 patients was not possible because of pTis status (no invasive tumor was found)

^a^In case of breast-conserving surgery (*n* = 264), in 0 patient reasons for direct reconstruction were reported. In case of modified radical mastectomy (*n* = 103), in 29 patients reasons for immediate breast reconstruction were reported

### Reporting of guideline recommendations with multiple alternatives

The CDTs for indication PST and IBR led to “leaves” recommending multiple alternatives. Regarding PST, the CDTs should have led to the alternatives “surgery first” or “PST ± MRI” in 171 (43.4%) patients. In 55 (32.2%) of these 171 patients, the MDT reported both alternatives. Regarding IBR, the CDTs should have led to the alternatives of surgery with or without IBR in 103 (28.1%) patients with MDT recommendation for modified radical mastectomy. In these 103 patients, the MDT reported IBR to be recommended (*n* = 18), to be considered (*n* = 6) and not to be recommended explicitly because high risk for postoperative radiation therapy (n = 5). In 74 of 103 patients (71.8%), the MDT did not document any information about the (im)possibility of IBR.

### Concordance of recommendations

The concordance rates between the recommendation “recommended” or “recommended for consideration” by the CDTs versus the recommendation generated by the MDT in patients of whom all data-items per CDT were available were 98%, 67%, 98% and 4% for the CDTs MRI scan, PST, AST and IBR, respectively (Table 3). In non-concordant cases, motivations for guideline deviation were not reported in 2%, 27%, 0% and 91% of cases, respectively.

**Table 3:** Concordance of recommendations generated by the MDT versus the CDTs in patients of which all data-items were available during MDT meetings

Recommendation	Patients		Concordant		Not concordant			
	*N*	%	*N*	%	Reasons not documented		Reasons documented	
					*N*	%	*N*	%
*MRI scan*	276	70						
Recommended/for consideration	49	18	48	98	1	2	NA	NA
Not recommended	227	82	6	3	219	96	2*	1
*PST*	52	13						
Recommended/for consideration	49	94	33	67	13	27	3**	6
Not recommended	3	6	0	0	3	100	NA	NA
*AST*	356^a^	97						
Recommended/for consideration	257	72	253	98	NA	NA	4^b^	2
Not recommended	98	28	91	93	NA	NA	7^c^	7
*IBR*	46	13						
Recommended	28	61	2	7	24	86	2^d^	7
For consideration	18	39	0	0	18	100	NA	NA

*Two patients received preoperative systemic treatment with preference to omit surgery in case of response to preoperative systemic therapy

**In three patients, preoperative systemic therapy was reported as an alternative in the electronic health record

^a^In one patient, the sentinel node procedure did not identify the sentinel node, and no pN status was available

^b^Three patients deliberately decided not to start adjuvant systemic treatment

^c^Seven patients were referred to the oncologist for the reason of "border-line" indication for adjuvant systemic treatment

^d^In two patients, the MDT did not recommend immediate breast reconstruction because irradiation of the thoracic wall was idicated

## Discussion

We found a low availability of data required for guideline-based recommendations at the time of decision-making. Complete availability and reporting of these data is important for generating verifiable guideline-based recommendations, especially when guidelines becoming more complex and patients are more involved in the decision-making process. In cases where the CDTs resulted in a guideline recommendation that consisted of multiple alternatives, these alternatives were reported by the MDT in only a minority of patients. MDT reporting of clear and motivated recommendations is valuable for internal communication between the different practitioners in the hospital and the patient. Further, we found high concordance rates between recommendations generated by the CDTs and the MDTs regarding indication for MRI scan and AST, but low rates regarding indication for PST and IBR.

In two out of four CDTs under study, we observed low percentages of data completeness in the electronic health record, mainly due to underreporting of “clinical M-stage” and “assessable mammography”. One might speculate that data-items can be assumed as known by the MDT but not explicitly reported (e.g., clinical M-stage). Our observation that an absent clinical phenomenon (actually “cM0”) is not reported by the MDT has been described earlier [7, 8]. For adequate CDT usage, it is however essential that all data-items are explicitly available to reach a “leaf” containing a guideline-based recommendation. Another reason for not reporting a specific item might be a lack of clear definition of that data-item. For example, “assessable mammography” was not described in uniform terms making a classification according to the ACR BI-RADS® criteria impossible in 112 (28%) patients [9]. In this particular case, this illustrates the need for adherence by radiologists to an appropriate definition and subsequent high-quality file management [10]. In general, completeness of data-items in the electronic health record can be improved if free text reporting is replaced by clearly defined standardized reporting of data-items [7, 11–13]. Further, standardized reporting, including clinical auditing, can be used to improve guideline compliance and to evaluate reasons for non-adherence [14–16].

Literature about documentation of multiple treatment alternatives in MDT reports in case the guideline recommendation includes more than one alternative is limited [17]. This is remarkable because the first steps in practicing informed decision-making are being aware that you have a choice and know the appropriate alternatives [18]. Hahlweg et al. analyzed 249 cases in 11 different cancer-specific MDT meetings and found that in 10% of cases more than one treatment recommendation was reported and this is comparable with our findings [17]. Explicit reporting the preferable timing of systemic therapy for early breast cancer, i.e., preoperative versus adjuvant, is done in only a small number of patients [19]. For IBR, it has already been shown that patients feel significantly more involved in shared decision-making if they are informed about the treatment alternatives [20].

There may be several reasons why MDTs do not report multiple alternatives when mentioned in the guideline recommendation. First, MDTs can guide the choices of the patients in a restrictive manner when they believe that alternatives are not equivalent and they have a clear preference, e.g., a patient with a tumor that can evidently be treated with breast-conserving surgery is unlikely to get a MDT recommendation including the alternative of mastectomy. Further, MDT members can consider factors that are not reported, e.g., the specific wish of a patient for a certain treatment or comorbidity of a patient making one alternative much more preferable above another [21]. Third, there may be internal agreements that in certain circumstances a particular alternative is not chosen, e.g., no PST in endocrine-sensitive early-stage breast cancer or a certain alternative may not be (timely) available in the local hospital, e.g., IBR. And finally, a reason may be that not all discussed alternatives by the MDT are reported.

The concordance of recommendations generated by the CDTs and the MDTs for indication of PST and IBR was low. There may be good reasons for not concordant cases. However, we found very low reporting rates for motivated deliberately guideline deviations, possibly by the lack of (time for) structured and systematically file management facilitating explicit motivations for MDT recommendations. CDTs deliver a systematical method to assess what treatment and diagnostic modalities are recommended according to the guideline. If we want to learn from real-world data, proper patient file management of relevant data-items and reasons for deliberately chosen alternatives or guideline deviations is an essential key. CDTs can be used to explicate the decision-making process, provided that all data-items are unambiguously present. In this way, CDTs act as a learning health system facilitating tightening and updating guidelines. Integrating learning health system data with existing knowledge from the literature can help to close the evidence-to-practice gap [22, 23].

The strength of our study was that two researchers independently evaluated the availability of data during MDT meetings and that all data-items were available from the electronic health records. Moreover, the cohort was representative for the Dutch population. The retrospective use of real-world data has the advantage that MDT participants were not influenced in their reporting manner (no Hawthorne effect). The retrospective manner is also a weak point as it is not verifiable if absent data-items or treatment alternatives mean that these were not considered/discussed following the CDT or not reported only. We found a lower percentage of Her2-positive breast cancers (4%), as to be expected in the Dutch population (13%) although Her2 status was available in 95% of cases [24]. However, we do not believe that this lower percentage biased our research objective. Further, we investigated the CDTs for only four clinical decisions in a single center, reflecting 15 unique data-items. It cannot be stated whether an availability of 13% of a data-item is exceptional or not. However, we found high availability rates of pathology data-items, and pathology data-items reflect 49% of all data-items in the guideline (56/114) [4].

## Conclusion

The availability of data in patient electronic health records that are essential for guideline-based recommendations as well as reporting of possible treatment alternatives of the CDTs under study was low. For meeting the conditions of EUSOMA, it is warranted that MDTs explicate information that is supposed to be implicitly known and to adhere to clear definitions of data-items in their reporting. Filling in the CDTs manually is time consuming and requires dedicated support from a nurse or data manager. For real-time use of CDTs in clinical practice, it is essential key that the needed data are registered in a standardized way, are exchangeable and reusable with MDT reporting forms and the CDTs. We recommend a prospective multicenter feasibility trial to observe if the data needed for CDT application is verbally or digital available during MDT meetings, distinguishing non-availability of data due to not being discussed or not being registered only.

## Abbreviations

AST: Adjuvant systemic treatment
CDT: Clinical decision tree
EUSOMA: European Society of Breast Cancer Specialists
IBR: Immediate breast reconstruction
MDT: Multidisciplinary team
MRI: Magnetic resonance imaging
PST: Preoperative systemic treatment
TNM: Tumor (T), nodes (N) and metastases (M)
